# SELF-REPORTED EVERYDAY DISCRIMINATION AND CARDIOMETABOLIC RISK IN OLDER BLACK AND WHITE ADULTS

**DOI:** 10.1093/geroni/igae098.1307

**Published:** 2024-12-31

**Authors:** Ryon Cobb

**Affiliations:** Rutgers, The State University of New Jersey, New Brunswick, New Jersey, United States

## Abstract

This study investigates the relationship between self-reported instances of everyday discrimination and cardiometabolic risk among older, focusing on potential variations by racial classification. This analysis draws on a subsample of older Black and White respondents from the 2010/2012 Health and Retirement Study (N=8,924), a biennial nationally representative survey of older adults across the United States. Cardiometabolic risk is assessed using a count of high-risk cardiometabolic biomarkers while we self-reported everyday discrimination and racial identification. We included an interaction term (self-reported everyday discrimination x race) to test whether the relationship between everyday discrimination and cardiometabolic risk varied by racial classification. We used negative binomial regression to estimate incidence rate ratios (IRRs) and corresponding 95% confidence intervals (CI) of self-reported everyday discrimination, racial classification, and cardiometabolic risk, controlling for potential confounders. Respondents with higher levels of self-reported everyday discrimination scores had higher levels of cardiometabolic risk after adjusting for demographic characteristics (IRR: 1.08, 95% C.I. 1.05-1.11), and while attenuated, remained significant (IRR: 1.08, 95% C.I. 1.01-1.07) after further adjustments for smoking status, physical activity, alcohol consumption, educational attainment, and household wealth. The relationship between self-reported instances of everyday discrimination and cardiometabolic risk was significantly weaker for older Black adults than their White counterparts. The present study confirms that self-reported instances of everyday discrimination are a psychosocial factor associated with higher levels of cardiometabolic risk among older adults; this association is especially pronounced among older White adults. Future studies among this population must examine whether the relationship changes over time.

